# Derivation and validation of a two-biomarker panel for diagnosis of ARDS in patients with severe traumatic injuries

**DOI:** 10.1136/tsaco-2017-000121

**Published:** 2017-08-28

**Authors:** Lorraine B Ware, Zhiguo Zhao, Tatsuki Koyama, Ryan M Brown, Matthew W Semler, David R Janz, Addison K May, Richard D Fremont, Michael A Matthay, Mitchell J Cohen, Carolyn S Calfee

**Affiliations:** 1 Department of Medicine, Vanderbilt University School of Medicine, Nashville, Tennessee, USA; 2 Department of Pathology, Microbiology and Immunology, Vanderbilt University School of Medicine, Nashville, Tennessee, USA; 3 Department of Biostatistics, Vanderbilt University School of Medicine, Nashville, Tennessee, USA; 4 Department of Medicine, Section of Pulmonary/Critical Care and Allergy/Immunology, Louisiana State University School of Medicine in New Orleans, New Orleans, Louisiana, USA; 5 Department of Surgical Science, Vanderbilt University School of Medicine, Nashville, Tennessee, USA; 6 Department of Medicine, Meharry Medical College, Nashville, Tennessee, USA; 7 Departments of Medicine and Anesthesia and the Cardiovascular Research Institute, University of California, San Francisco, California, USA; 8 Department of Surgery, Denver Health Medical Center, Denver, Colorado, USA; 9 University of Colorado, Aurora, Colorado, USA

**Keywords:** acute lung injury, ARDS, endothelium, lung epithelium, trauma

## Abstract

**Background:**

Acute respiratory distress syndrome (ARDS) is common after severe traumatic injuries but is underdiagnosed and undertreated. We hypothesized that a panel of plasma biomarkers could be used to diagnose ARDS in severe trauma. To test this hypothesis, we derived and validated a biomarker panel in three independent cohorts and compared the diagnostic performance to clinician recognition of ARDS.

**Methods:**

Eleven plasma biomarkers of inflammation, lung epithelial and endothelial injury were measured in a derivation cohort of 439 severe trauma patients. ARDS status was analyzed by two-investigator consensus, and cases were required to meet Berlin criteria on intensive care unit (ICU) day 1. Controls were subjects without ARDS during the first 4 days of study enrollment. A multivariable logistic regression model was used to generate probabilities for ARDS. A reduced model with the top two performing markers was then tested in two independent validation cohorts. To assess clinical diagnosis of ARDS, medical records in the derivation cohort were systematically searched for documentation of ARDS diagnosis made by a clinical provider.

**Results:**

Among 11 biomarkers, the combination of the endothelial injury marker angiopoietin-2 (Ang-2) and the lung epithelial injury marker receptor for advanced glycation endproducts (RAGE) provided good discrimination for ARDS in the derivation cohort (area under the curve (AUC)=0.74 (95% CI 0.67 to 0.80). In the validation cohorts, the AUCs for this model were 0.70 (0.61 to 0.77) and 0.78 (0.71 to 0.84). In contrast, provider assessment demonstrated poor diagnostic accuracy for ARDS, with AUC of 0.55 (0.51 to 0.60).

**Discussion:**

A two-biomarker panel consisting of Ang-2 and RAGE performed well across multiple patient cohorts and outperformed clinical providers for diagnosing ARDS in severe trauma. Clinical application of this model could improve both diagnosis and treatment of ARDS in patients with severe trauma.

**Level of evidence:**

Diagnostic study, level II.

## Introduction

Acute respiratory distress syndrome (ARDS) is common in patients with severe traumatic injuries.^1^ Although effective therapies for ARDS are limited,^2^ timely recognition of ARDS and treatment with a lung protective mechanical ventilation strategy improves mortality.^3 4^ In subgroups of patients with more severe ARDS or those with absence or resolution of shock, therapies such as prone positioning^5^ or conservative fluid therapy^6^ may also be beneficial. However, the clinical diagnosis of ARDS is frequently delayed or missed,^7 8^ contributing to belated, inadequate or inappropriate treatment.

Plasma biomarkers are commonly used in conditions such as myocardial infarction and congestive heart failure to aid in clinical diagnosis and facilitate rapid application of appropriate therapies. Although plasma biomarkers of inflammation, lung epithelial and endothelial injury may facilitate early identification of patients with ARDS,^9 10^ the utility of plasma biomarkers for diagnosis of ARDS in patients with traumatic injuries has not been systematically investigated in large cohorts of patients. Only one prior study has examined plasma biomarkers for ARDS diagnosis among trauma patients, a small, single-center case–control study limited by discordant timing of plasma sampling and ARDS diagnosis.^11^

To rigorously examine the diagnostic performance of plasma biomarkers for ARDS among adult trauma patients, we evaluated 11 previously described biomarkers of inflammation and epithelial or endothelial injury in three prospective observational cohorts of patients with severe traumatic injury. We hypothesized that a panel of plasma biomarkers could accurately identify trauma patients with ARDS and that a panel of plasma biomarkers would improve ARDS diagnosis compared with clinician recognition alone.

## Methods

### Patient cohorts and clinical data collection

The derivation cohort consisted of 439 consecutive patients with severe traumatic injuries requiring admission to the Vanderbilt trauma intensive care unit (ICU) who were enrolled prospectively in the Validating Acute Lung Injury biomarkers for Diagnosis (VALID) cohort at Vanderbilt University Medical Center between 2006 and 2009. The VALID cohort is a prospective observational cohort study of critically ill patients at risk for ARDS. Inclusion criteria, enrollment and consent procedures have been previously described,^12^ and the VALID study was approved by the Vanderbilt Institutional Review Board (IRB) with a waiver of informed consent if the patient or surrogate were unable to provide consent. Patients were enrolled on the morning of ICU day 2 and phenotyped for ARDS daily for the first four ICU days. Within this cohort, cases (n=78) were defined as ARDS if they met Berlin ARDS criteria^13^ on ICU day 1 by review of clinical data and chest radiographs by consensus of two physician investigators who were blinded to biomarker levels. Controls were all other subjects (n=315) who did not meet Berlin ARDS criteria during the first 4 days of study enrollment. Patients who only met ARDS criteria on a single day or who first met criteria on ICU day 2, 3 or 4 (n=46) were excluded from the primary analysis but included in two sensitivity analyses first as cases, then as controls.

The primary validation cohort consisted of 200 patients with severe blunt traumatic injuries enrolled in the University of California, San Francisco (UCSF) Activation of Coagulation and Inflammation in Trauma (ACIT) cohort at San Francisco General Hospital between 2005 and 2013.^14^ Detailed inclusion and exclusion criteria have been previously described.^14^ The study was approved by the UCSF Committee on Human Research with deferred informed consent, as previously described.^14^ In the ACIT cohort, patients were enrolled on arrival to the emergency department. ARDS was defined on the basis of Berlin criteria and ascertained by a two-physician review of all patient data for the first 7 days of hospitalization. For this analysis, cases were defined as ARDS if they met Berlin ARDS criteria^13^ within 48 hours of ICU admission by review of clinical data and chest radiographs by a two-physician investigator consensus. Controls were all other subjects without ARDS during the first 7 days of study enrollment. Patients who developed ARDS beyond 48 hours of ICU admission (n=29) were excluded from the primary analysis. Six patients with indeterminate ARDS status due to missing data were excluded from all analyses.

As an additional validation study, the final two-biomarker model for diagnosis of ARDS was subsequently tested using existing clinical data and biomarker levels^11^ from 190 patients with severe traumatic injuries. These patients were enrolled in a separate Vanderbilt IRB-approved study examining the impact of gender on ICU infection and mortality in patients with severe trauma or surgical critical illness, referred to here as the gender study.^15^ This group of patients included 74 control patients with clear chest radiographs during the first 7 days of hospitalization, 11 control patients with clinical evidence of hydrostatic pulmonary edema and 105 case patients with ARDS (defined by Berlin criteria) within 72 hours of ICU admission.^11^

### Plasma biomarker measurements

For the derivation study, biomarkers were selected based on published studies of biomarkers for diagnosis and prognosis in ARDS and were chosen to reflect pathways that are commonly implicated in the pathogenesis of acute lung injury including inflammation, coagulation, and lung epithelial and endothelial injury. All biomarkers were measured in duplicate in plasma that was collected at enrollment on the morning of ICU day 2, using commercially available enzyme immunoassay kits or radioimmunoassay: plasminogen activator inhibitor-1, American Diagnostica (Stamford, CT); von Willebrand factor antigen (VWF), Diagnostica Stago (Parsippany, NJ); procollagen peptide-III, Cis Bioscience International (Bedford, MA); club cell-16 protein (CC16), BioVendor (Chandler, NC); B-type natriuretic peptide, Bachem Bioscience (King of Prussia, PA); surfactant protein-D (SP-D), Yamasa Corporation (Tokyo, Japan); and angiopoietin-2 (Ang-2), interleukin-8 (IL-8), soluble receptor for advanced glycation endproducts (RAGE), R&D Systems (Minneapolis, MN). For the ACIT validation cohort, Ang-2 and RAGE were measured in plasma that was collected 24 hours after presentation to the emergency department using the assay kits from the same manufacturers. For the gender study cohort, Ang-2 and RAGE levels that had been previously measured using kits from the same manufacturers^11^ were used.

### Clinician recognition of ARDS

To compare the performance of the biomarker panel for diagnosis of ARDS to clinician recognition of ARDS, the electronic medical record of each patient in the derivation cohort was systematically searched for documentation of a diagnosis of ARDS during the hospitalization by a clinical provider by scanning for key words including ‘acute lung injury’, ‘acute respiratory distress syndrome’, ‘ALI’ and ‘ARDS’. Prior to implementation, the accuracy of the electronic search algorithm was confirmed by manual review of all history, physical and progress notes for 100 patients in the derivation cohort. In addition, all positive results for key words from the electronic search were corroborated by investigator review of the relevant documents to confirm that a clinical diagnosis of ARDS was present or suspected.

### Statistical analysis

Demographic and clinical variables were analyzed within each study cohort using Wilcoxon rank-sum test for continuous variables and Pearson’s χ^2^ test for categorical variables. Biomarker values underwent logarithmic transformation to reduce right skewness. When measured biomarker levels were below the assay detection limit, a value was imputed at half the lower limit of detection for each biomarker. In the derivation cohort, we used a backward elimination model-building strategy on 1000 bootstrapped datasets to select the biomarkers for further consideration in a logistic regression model. For each bootstrap sample, a full model with all 11 biomarkers was fit. Then, the biomarker with the largest p value (Wald test) was dropped, and a new model was fit with one fewer biomarker. This backward elimination process was repeated until only one biomarker was retained. The biomarkers were then ranked from most significant (the last one to remain) to least significant (the first one eliminated) and the average rank from 1000 bootstrap repetitions was used to select the variables for further consideration. The full 11-biomarker logistic model, and the best two-biomarker (Ang-2 and RAGE) logistic model were then fit. The optimism of the model was evaluated by a 300-iteration bootstrap validation. The performance of the model was measured using receiver operating characteristics curves and the area under the curve (AUC) and compared with the clinician diagnosis of ARDS. The two-biomarker model was used to compute the ORs and 95%CI for ARDS.

To externally validate the two-biomarker model, we fixed the coefficients of the model and computed the predicted probabilities of ARDS in the two cohorts. The discrimination ability of the model was evaluated in each validation cohort, using the Harrell C-statistic. The 95% CIs for the C-statistic were generated with 300 bootstrap samples.

Two sensitivity analyses were conducted in the VALID cohort that included 46 patients who were initially excluded from analysis, those patients who had delayed onset of ARDS (met ARDS criteria on day 2 or later). These patients were included first as ARDS cases then as controls in sensitivity analyses. All analyses were performed with R V.3.3 (R Foundation for Statistical Computing, Vienna, Austria).

## Results

### Patients

Patient characteristics are summarized in table 1. Across the three patient cohorts, patients with and without ARDS were similar in terms of age, gender and race/ethnicity. Compared with subjects without ARDS, subjects with ARDS had significantly higher injury severity scores and Acute Physiology and Chronic Health Evaluation (APACHE) II scores, along with higher hospital mortality and longer duration of mechanical ventilation.

**Table 1:** Patient characteristics by patient cohort

	VALID cohort			ACIT cohort			Gender study cohort		
	ARDS (n=78)	No ARDS (n=315)	p	ARDS (n=75)	No ARDS (n=119)	p	ARDS (n=105)	No ARDS (n=85)	p
Age (years)	42 (26–55)	41 (27–55)	0.79	41 (27–59)	40 (27–60)	0.93	39 (25–53)	33 (23–47)	0.18
Female	22 (17)	27 (85)	0.35	16 (12)	29 (356)	0.03	34 (36)	29 (25)	0.50
Caucasian	78 (61)	80 (252)	0.94	73 (55)	63 (75)	0.14	83 (87)	84 (71)	0.31
Blunt trauma	86 (67)	84 (263)	0.73	100 (75)	100 (119)	NA	85 (88)	86 (72)	0.96
TBI	50 (39)	58 (182)	0.25	75 (56)	71 (84)	0.53	36 (37)	51 (43)	0.036
Arterial base deficit (mEq/L)	8.8±5.0	6.0±5.1	<0.001	6.8±6.5	4.0±4.9	0.007	7.2±5.8	4.6±4.2	<0.001
PaO_2_/FiO_2_	143 (103–190)	251 (164–337)	<0.001	244 (143–380)	349 (212–454)	0.003	72 (38–140)	123 (89–153)	<0.001
ISS	33 (26–38)	26 (18–34)	<0.001	30 (26–43)	26 (14–30)	<0.001	34 (25–41)	29 (17–35)	0.002
APACHE II	27 (22–31)	22 (18–27)	<0.001	16 (12–20)	17 (8–23)	0.84	17 (14–21)	13 (11–17)	<0.001
Vent days (to 28 days)	10 (4–15)	4 (2–9)	<0.001	11 (7–21)	3 (2–8)	<0.001	9 (5–14)	4 (3–7)	<0.001
Hospital mortality	10 (8)	9 (29)	0.78	36 (27)	14 (17)	<0.001	14 (15)	5 (4)	0.03
Ang-2 (pg/mL)	5880 (4429–7724)	4007 (2763–5816)	<0.001	6812 (4323–12223)	4393 (3093–6378)	<0.001	8681 (5724–14044)	5697 (3784–8521)	<0.001
RAGE (pg/mL)	1886 (956–3298)	944 (646–1523)	<0.001	1184 (847–2068)	912 (588–1348)	<0.001	2505 (1779–5081)	1415 (1024–2022)	<0.001

Data as mean±SD, median (IQR) or percentage (number).

ACIT, Activation of Coagulation and Inflammation in Trauma; Ang-2, angiopoietin-2; APACHE II, Acute Physiology and Chronic Health Evaluation II; FiO_2_, fractional inspired oxygen; ISS, injury severity score; ARDS, acute respiratory distress syndrome; NA, not available; PaO_2_, arterial oxygen tension (or pressure); RAGE, receptor for advanced glycation endproducts; TBI, traumatic brain injury, defined as AIS head ≥3; VALID, Validating Acute Lung Injury biomarkers for Diagnosis.

### Derivation of a biomarker model for diagnosis of ARDS

**Figure 1:**
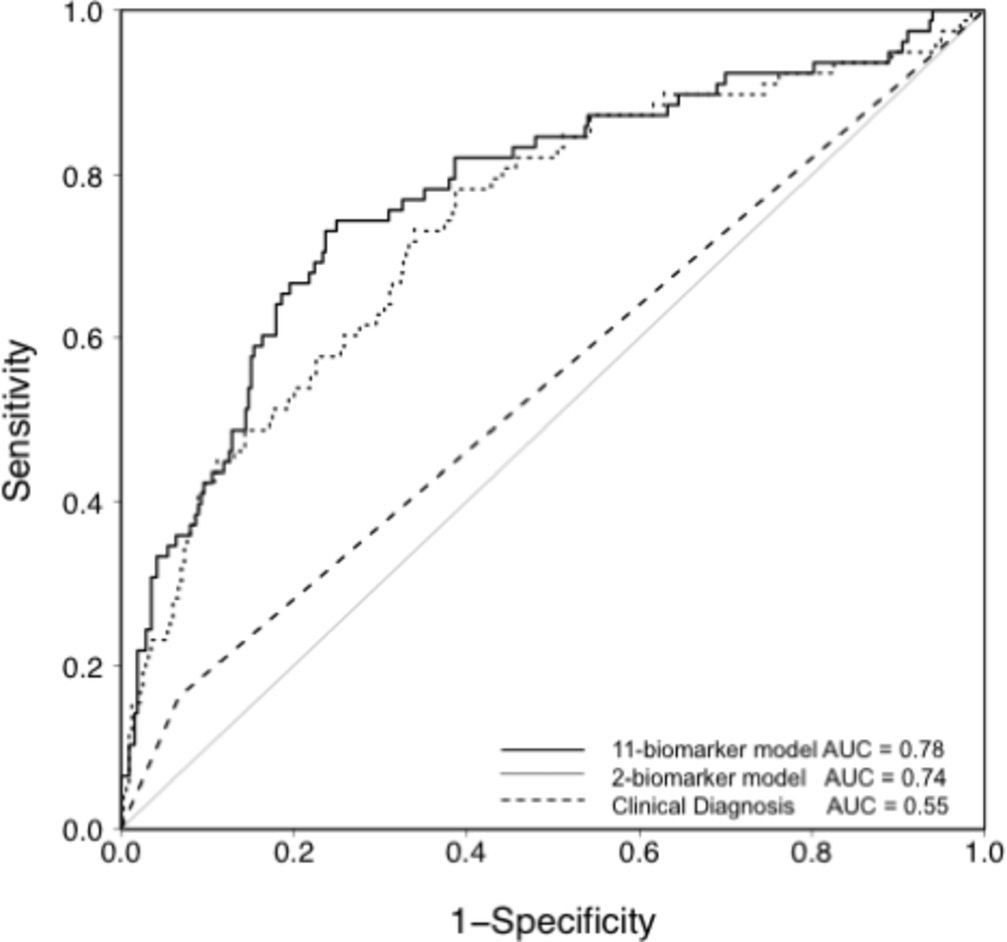
Receiver operator characteristic (ROC) curve analysis of the plasma biomarker panels for differentiating ARDS (cases) from controls in patients with severe traumatic injuries in the VALID cohort. The solid line shows predicted probability of occurrence of ARDS for each subject computed from a logistic regression model that included 11 biomarkers (RAGE, PCPIII, BNP, Ang-2, IL-8, TNF-α, IL-10, VWF, SP-D, PAI-1 and CC16). Specificity and sensitivity were computed at each possible cut-off of the predicted probability. The area under the curve (AUC) is 0.78. The dotted line shows the ROC analysis in the same patients using only the two most discriminatory biomarkers (RAGE and Ang-2). The AUC for this model is 0.74. For comparison, the dashed line shows the ROC analysis for clinician recognition of ARDS with an AUC of 0.55. Ang-2, angiopoietin-2; BNP, brain natriuretic peptide; CC16, club cell-16 protein; IL-8, interleukin 8; IL-10; interleukin 10; PAI-1, plasminogen activator inhibitor-1; PCPIII, procollagen peptide-III; RAGE, receptor for advanced glycation endproducts; SP-D, surfactant protein-D; TNF-α, tumor necrosis factor-α; VALID, Validating Acute Lung Injury biomarkers for Diagnosis; VWF, von Willebrand factor antigen.

**Figure 2:**
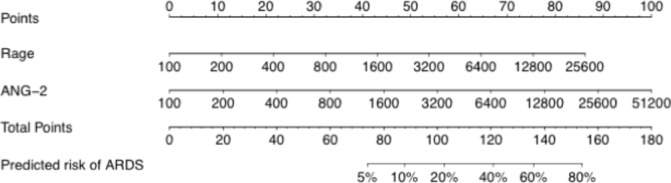
The two biomarker (RAGE and Ang-2) multivariable logistic regression model was used to create a prediction model for the probability of ARDS. The value for each predictor variable (RAGE, Ang-2, both in pg/mL) is used to determine a number of points using the point scale at the top. The sum of the individual predictor variable points for the measured RAGE and Ang-2 levels corresponds to the total points and the probability of ARDS shown at the bottom. Ang-2, angiopoietin-2; ARDS, acute respiratory distress syndrome; RAGE, receptor for advanced glycation endproducts.

Levels of the 11 plasma biomarkers in the derivation cohort are shown in table 2. Of the 11 biomarkers, five were significantly different between cases and controls in univariate analysis: Ang-2, RAGE, IL-8, VWF and CC16. A logistic regression model was fit for diagnosis of ARDS using all 11 biomarkers, and the model performance was assessed by the area under the receiver operator characteristic curve (AUC). As shown in figure 1, the AUC for the 11-biomarker panel was 0.78 (95% CI 0.73 to 0.85). To minimize overfitting and maximize the potential for rapid bedside measurement, a second model was tested that used only the two top-performing biomarkers (Ang-2 and RAGE). This model is summarized in table 3. The AUC for the two-biomarker panel was 0.74 (95% CI 0.67 to 0.80) (figure 1). To illustrate how the two-biomarker panel might be applied at the bedside for ARDS diagnosis, a nomogram for the model is shown in figure 2.

**Table 2:** Comparison of 11 plasma biomarkers between ARDS cases and controls in the derivation cohort

Biomarker	ARDS (n=79)	No ARDS (n=318)	p Value
RAGE (pg/mL)	1886 (956–3298)	944 (646–1523)	**<0.001**
PCPIII (ng/mL)	3.9 (2.9–5.0)	3.5 (2.7–4.8)	0.251
BNP (ng/mL)	0.4 (0.3–0.7)	0.4 (0.3–0.6)	0.312
Ang-2 (pg/mL)	5880 (4429–7724)	4007 (2763–5816)	**<0.001**
IL-8 (pg/mL)	15.6 (15.6–57.7)	15.6 (15.6–34.9)	**0.017**
TNF-α (pg/mL)	1.0 (0.6–3.0)	1.4 (0.6–5.1)	0.195
IL-10 (pg/mL)	18.2 (9.4–82.4)	18.1 (8.1–56.7)	0.373
VWF (% control)	230 (173–353)	270 (198–364)	**0.045**
SP-D (ng/mL)	60.3 (37.2–91.9)	53.6 (32.9–78.4)	0.061
PAI-1 (ng/mL)	118.9 (39.1–248.5)	92.3 (50.6–174.5)	0.476
CC16 (ng/mL)	7.0 (4.2–11.2)	5.5 (3.4–8.6)	**0.004**

Data as median (IQR). Ang-2, angiopoietin-2; BNP, brain natriuretic peptide; CC16, club cell-16 protein; IL-8, interleukin 8; IL-10; interleukin 10; PAI-1, plasminogen activator inhibitor-1; PCPIII, procollagen peptide-III; RAGE, receptor for advanced glycation endproducts; SP-D, surfactant protein-D; TNF-α, tumor necrosis factor-α; VWF, von Willebrand factor antigen.

**Table 3:** Summary of the two-biomarker model for diagnosis of ARDS in the derivation cohort

Biomarker (upper and lower quartiles)	OR for ARDS*	95% CI	p
RAGE (1846 vs 656)	2.382	1.638 to 3.464	<0.001
Ang-2 (6128 vs 2935)	1.890	1.322 to 2.702	<0.001

*OR for ARDS comparing upper quartile (75th percentile) to lower quartile (25th percentile).

Ang-2, angiopoietin-2; ARDS, acute respiratory distress syndrome; RAGE, receptor for advanced glycation endproducts.

### Sensitivity analyses

Because patients who had either delayed development of ARDS (after ICU day 1) were excluded from the initial model derivation, two sensitivity analyses were conducted. In the first sensitivity analysis, the 46 excluded patients were included as ARDS cases and 11-biomarker and two-biomarker logistic regression models were constructed; model performance was reduced with AUCs of 0.72 (95% CI 0.69 to 0.79) for the 11-biomarker model and 0.67 (95% CI 0.63 to 0.73) for the two-biomarker model (likelihood ratio test, p=0.017). By contrast, in the second sensitivity analysis when the 46 excluded patients were included as non-ARDS controls, model performance was very close to the original derivation with AUC of 0.77 (95% CI 0.73 to 0.84) for the 11-biomarker model and 0.74 (95% CI 0.67 to 0.80) for the two-biomarker model (likelihood ratio test, p=0.071).

### Validation of the two-biomarker model in the ACIT cohort

Performance of the two-biomarker model using Ang-2 and RAGE was validated in a separate cohort of 165 patients with severe traumatic injuries who were enrolled in the ACIT study. Performance of the two-biomarker model for diagnosis of ARDS was slightly lower in the ACIT cohort (AUC 0.70, 95% CI 0.61 to 0.77) compared with the derivation cohort.

### Validation of the two-biomarker model in the gender study cohort

Performance of the two-biomarker model was also validated in a separate cohort of 190 patients with severe traumatic injuries who were enrolled in a study of gender and ICU-acquired infections. Performance of the two-biomarker model for diagnosis of ARDS was excellent in the gender study cohort (AUC 0.78, 95% CI 0.71 to 0.84). Of note, this cohort included patients with both clear chest radiographs and evidence of hydrostatic pulmonary edema in the control group.

### Comparison of the biomarker model to clinician diagnosis in the VALID cohort

To analyze whether the two-biomarker panel outperformed clinician recognition of ARDS, the electronic medical record of each patient in the derivation cohort was systematically scanned for documentation of a diagnosis of ARDS during the hospitalization by a clinical provider. The two-biomarker panel significantly outperformed (Akaike Information Criterion reduced by 25) clinician recognition which had an AUC of 0.55 (95% CI 0.51 to 0.60, figure 1), and a sensitivity of 16% and specificity of 93% when compared with the gold standard diagnosis by two investigator consensus.

## Discussion

Although there have been many studies of the prognostic value of biomarkers in patients with established ARDS,^16 17^ there are relatively few studies of the diagnostic value of plasma biomarkers in ARDS. The primary goal of this study was to derive and validate a plasma biomarker panel for diagnosis of ARDS in adult patients with severe traumatic injuries. We tested 11 biomarkers of various aspects of the pathophysiology of ARDS including biomarkers of inflammation, lung epithelial injury, endothelial injury and coagulation. To minimize overfitting and maximize the potential for rapid bedside application in the future, this panel was narrowed to the top two performing biomarkers and then validated in two independent patient cohorts. The two-biomarker panel of Ang-2 and RAGE performed similarly across the three cohorts and significantly outperformed clinical diagnosis.

Although it has been reported that ARDS is underdiagnosed and undertreated,^3 7 8 18^ the current study underscores the potential magnitude of this problem. In a detailed review of all history and progress notes for each patient in the derivation cohort, we found that ARDS was largely undiagnosed; clinician recognition had a sensitivity of only 31% for the presence of ARDS, with an AUC of 0.55. Thus, although performance of the two-biomarker panel is only in the moderate range for a diagnostic test, when the performance of the two-biomarker panel is compared with clinician recognition of ARDS, there is clear value. Since timely diagnosis and adherence to low tidal volume ventilation decreases ICU mortality,^3^ utilization of this simple biomarker panel could expedite diagnosis and treatment of ARDS in severe trauma, leading to improved clinical outcomes. In addition, application of the two-biomarker panel could facilitate identification of patients for inclusion in clinical trials of new therapies for trauma-associated ARDS.

Although we tested biomarkers of many aspects of the pathophysiology of ARDS including inflammation, activation of fibrotic pathways, dysregulated coagulation and a biomarker of increased intravascular volume, the top two performing biomarkers that make up the final two-marker panel are biomarkers of lung epithelial injury (RAGE) and endothelial injury (Ang-2). Both lung epithelial injury and endothelial injury are key features of ARDS that have been well documented in both experimental and clinical acute lung injury, lending biologic plausibility to the findings. Lung epithelial injury is a pathophysiologic hallmark of ARDS^19^ that is evident both pathologically^20^ and in functional assays of alveolar epithelial transport function.^21^ RAGE is highly expressed on the type I alveolar epithelium^22^; release of RAGE into the airspace and circulation is a biomarker of lung epithelial injury in rodent,^22^ human experimental^23^ and clinical studies.^24^ In patients at risk for ARDS from sepsis, biomarkers of lung epithelial injury including RAGE, SP-D and CC16 are strongly associated with the diagnosis of ARDS,^9^ suggesting that biomarkers of lung epithelial injury may be useful for diagnosis of ARDS in patients with a variety of underlying risk factors beyond just patients with severe traumatic injuries.

Injury to the microvascular endothelium is also a pathophysiologic hallmark of ARDS.^2^ Several biomarkers of endothelial injury have previously been associated with development of ARDS including VWF^25^ and Ang-2. Ang-2, an endothelial growth factor and potent mediator of vascular permeability and endothelial injury, was recently found to be highly predictive of ARDS among patients presenting to the emergency department who were at risk for, but did not yet have, ARDS.^10^ This study did not include patients with traumatic injuries. The current study extends prior findings to patients with traumatic injuries and suggests that endothelial injury is a key pathophysiologic feature of trauma-induced ARDS that can be used to aid clinical diagnosis.

This study has several strengths. First, because of the large size of the derivation cohort, we were able to test a large number of potential biomarkers in the derivation cohort, and thus the study included candidate biomarkers of diverse aspects of the pathophysiology of ARDS. Second, all three of the cohorts that were used had dedicated ARDS phenotyping done by the study authors, including two physician review of all chest radiographs and clinical data, insuring the validity of the gold standard ARDS diagnosis for this study. Finally, validation of the biomarker panel in two independent cohorts enhances the external validity of the findings.

There are also some limitations. Due to differences in how the three cohorts were phenotyped for ARDS in the three parent studies, each cohort used slightly different definitions for cases and controls. This might, in part, explain why the biomarker panel performance varied somewhat between cohorts. In addition, the gender cohort was a case–control cohort, and the performance of the biomarker panel in this group may be an overestimate of panel performance due to patient selection. As noted above, the overall performance of the two-biomarker panel was only in the moderate range, although biomarkers significantly outperformed clinical diagnosis. Finally, since the studies were limited to patients with severe traumatic injuries, the findings cannot be generalized to other patient populations.

In summary, a two-biomarker panel consisting of Ang-2 and RAGE performed well across multiple patient cohorts and outperformed clinical providers for diagnosing ARDS in patients with severe traumatic injuries. If validated prospectively, clinical application of this model could improve both diagnosis and timely treatment of ARDS.
